# Pathogenic changes of community-acquired pneumonia in a children’s hospital in Beijing, China before and after COVID-19 onset: a retrospective study

**DOI:** 10.1007/s12519-022-00592-8

**Published:** 2022-08-22

**Authors:** Li-Na Zhang, Ling Cao, Ling-Hui Meng

**Affiliations:** 1 Department of Pulmonology, Children’s Hospital, Capital Institute of Pediatrics, Beijing, China; 2 Center for Evidence-Based Medicine, Office of Science and Technology, Capital Institute of Pediatrics, Beijing, China

**Keywords:** Community-acquired pneumonia, Comparison, Coronavirus disease 2019, Pathogen

## Abstract

**Background:**

This study aimed to analyze the pathogenic characteristics of community-acquired pneumonia (CAP) in a children’s hospital before and after the coronavirus disease 2019 (COVID-19) pandemic and to provide testimony for preventing CAP in the future.

**Methods:**

A retrospective analysis was performed. The information was collected from the electronic medical record system of the hospital. A total of 2739 children were included from February 1, 2019, to January 31, 2021.

**Results:**

Among these 2739 patients were 1507 (55.02%) males and 1232 (44.98%) females; the median age was 3.84 years. There were 2364 cases during the pre-COVID-19 period and 375 cases during the post-COVID-19 period. The number of hospitalized children after the pandemic was 84.14% lower. The median age after the onset was 1.5 years younger than that before the onset (4.08 years old) (*Z* = − 7.885, *P* < 0.001). After the pandemic, the proportion of CAP in school-age children and *Mycoplasma pneumoniae* pneumonia (MPP) and influenza virus pneumonia (IVP) decreased significantly. During the pre-COVID-19 period, the proportions of detected pathogens were as follows: MP (59.56%) > bacteria (50.42%) > viruses (29.57%) > fungi (3.43%). During the post-COVID-19 period, the pathogen proportions were bacteria (56.53%) > viruses (53.60%) > MP (23.47%) > fungi (3.73%).

**Conclusions:**

There was a significant decrease in the number of children with CAP hospitalized after the pandemic, especially among school-age children, and the pathogen proportions of CAP with MP and IV were significantly decreased. We inferred that CAP was effectively prevented in school-age children because of the strong mitigation measures.

## Introduction

Community-acquired pneumonia (CAP) is the leading cause of morbidity among childhood respiratory diseases. The World Health Organization (WHO) estimates that nearly one-fifth of childhood deaths are caused by pneumonia, which claims more than 2 million lives annually [1]. The global child mortality and causes of death analysis, published by the Child Health Epidemiology Expert Group of the World Health Organization and the United Nations Children's Fund, shows that infectious diseases accounted for 64% of deaths in children under five years of age worldwide in 2010. Among all infectious diseases, the mortality rate of pneumonia is 14.1%, especially in developing countries [2]. This not only consumes a large amount of medical resources and brings a serious economic burden to society and families but also seriously endangers children's health. After the coronavirus disease 2019 (COVID-19) pandemic, we found that the number of children hospitalized with CAP decreased markedly. This study was conducted in a children's hospital in Beijing, China and aimed to analyze the pathogenic characteristics of CAP before and after the onset of the pandemic and to provide testimony for preventing CAP in the future.

## Methods

A retrospective analysis was used in our study, which was conducted in a 400-bed children’s hospital in Beijing, China from February 1, 2019 to January 31, 2021. Children between ≥ 28 days and 18 years of age who completed pathogen testing within 48 hours after admission and met the diagnostic criteria for CAP were included in this study. Referring to “Zhu Futang’s Practice of Pediatrics” (8th edition), our diagnostic criteria [3] for CAP in children were as follows: (1) cough, expectoration, wheezing and dyspnea recently with or without chest pain or aggravation of the original respiratory symptoms; (2) fever; (3) signs of pulmonary consolidation and/or moist rales; and (4) patchy, infiltrative shadows or interstitial changes in the lung with or without pleural effusion in the chest X-ray. CAP was clinically diagnosed when the criteria for (1) or (2) were combined with the criteria for (3) or (4). Hospital-acquired pneumonia, chronic pneumonia, tuberculosis, tracheobronchial foreign bodies, aspiration pneumonia, parasitic lung disease, and diffuse pulmonary interstitial/parenchyma disease were excluded.

A total of 3728 children were diagnosed with pneumonia during the study period and admitted to our hospital, and 2739 cases were included according to the above exclusion criteria. The details are as follows: 63 cases were excluded because of a tracheobronchial foreign body, nine cases were excluded because of tuberculosis, four cases were excluded because of aspiration pneumonia, nine cases were excluded because of tuberculosis, 365 cases were excluded because of follow-up examination after treatment, 253 cases were excluded because of convalescence of pneumonia, 35 cases were excluded because the flexible bronchoscopy was checked, and 260 cases were excluded because of allergic pneumonia, interstitial lung disease or repeated recent hospitalization elsewhere before entering our hospital.

According to their age, children were divided into an infant group (≥ 28 days to < 1 year of age), a young age group (1 to < 3 years of age), a preschool group (3 to < 6 years of age), and a school-age group (≥ 6 years of age). For the season variable, the children were divided into a spring group (March 1–May 31), summer group (June 1–August 31), autumn group (September 1–November 30) and winter group (December 1–February 28). Since January 31, 2020, widespread and strict isolation measures have been implemented in Beijing; therefore, February 1, 2019, to January 31, 2020, was defined as the pre-COVID-19 period, and February 1, 2020, to January 31, 2021, was defined as the post-COVID-19 period.

Regarding specimen collection, sputum specimens were collected routinely from all children with CAP. Throat swabs were taken only for severe acute respiratory syndrome coronavirus-2 virus (SARS-CoV-2) nucleic acid. The clinician decided which specimen would be taken. Bronchoalveolar lavage fluid (BALF) was collected from children who had large areas of pulmonary consolidation. Pleural effusion samples were taken from children with a large amount of pleural effusion. (1) Sputum specimens: all patients’ mouths were washed with normal saline before the specimen was taken. Approximately 1–2 mL of sputum in the deep airway was absorbed through the nasal cavity (inserted 6–12 cm) under negative pressure. If the sample was not sufficient, it was resampled. Qualified sputum specimens included those with ≤ 10 squamous epithelial cells, ≥ 25 multinucleated leukocytes, or a ratio of the two cells < 1:2.5 per low-power microscope field view; (2) serological samples: a total of 2–5 mL of venous blood was collected from all patients within 48 hours after admission; (3) pharyngeal swab: the tongue was fixed with a tongue depressor, and a sterile cotton swab was placed into the pharynx, passed the root of the tongue to the posterior pharyngeal wall, tonsil recess and lateral wall, and wiped repeatedly 3–5 times to collect mucosal cells; (4) BALF specimen: flexible bronchoscopy was used to intubate the infected lung through the nasal cavity, and more than 5 mL of BALF was collected and sent for examination within 0.5 hours; (5) pleural effusion: according to the standard process of pleural puncture, at least 5 mL of puncture fluid was taken under sterile conditions for examination.

The pathogen detection steps were as follows: (1) fungal detection: suspicious fungal-infected patients were tested by blood for (1,3)-β-d-glucan detection (G test) and galactomannan (GM test), sputum or BALF for fungal culture; (2) antibody detection: a gelatin particle agglutination test was used to detect antibody titers (the kit was manufactured by Fujie Bio Inc., Japan). The antibodies against SARS-CoV-2 were tested using the direct chemiluminescence method (the “SARS-CoV-2 IgG and IgM antibody test kit” purchased from Maccura Biological Co., Ltd., USA); (3) bacterial culture: sputum, pleural effusion or BALF was collected and inoculated on petri dishes (automatic culture instrument purchased from BD, USA and sterile sputum culture bottles purchased from Oxoid, UK); (4) pathogen nucleic acid detection: the nucleic acid extract was extracted using the nucleic acid extraction QIAamp MinElute virus spin kit (Qiagen GmbH, Germany). The polymerase chain reaction (PCR) test (NxTAG™ RPP kit purchased from Luminex, Canada) or the thermostatic amplification chip method (the kit was purchased from Beijing Boao Biological Group Co., Ltd., Item No. 360090; SARS-CoV-2 nucleic acid test kit purchased from Zhongshan University Daan Gene Co. Ltd.) were used to detect nucleic acids.

Regarding CAP pathogen evaluation, all the following criteria were combined with clinical symptoms for a final diagnosis. The etiology is not equivalent to the mere detection of a pathogen. (1) Bacterial CAP: if the bacterial cultures or nucleic acid results were positive, clinical manifestations were combined to determine whether the test result was indicative of the corresponding bacterial infection or just colonization; (2) viral CAP: positive viral [respiratory syncytial virus (RSV), human metapneumovirus, influenza] DNA or RNA results indicated a viral infection, and others were diagnosed by clinicians after comprehensive consideration; (3) community-acquired *Mycoplasma pneumoniae* pneumonia (MPP) was measured as follows: 1) serum MP antibody ≥ 1:32, 2) serum MP antibody ≥ 1:160 and MP PCR test was positive, or 3) the MP antibody titer of the recovery phase and acute phase increased or decreased by 4 times or more; 4) fungal CAP: a hierarchical diagnosis method was adopted, including host risk factors and clinical, microbiological and histopathological evidence. The diagnosis was divided into three levels: confirmed diagnosis, clinical diagnosis and suspected diagnosis [4].

An electronic medical record system called EMR developed by the software company “Beijing Jiahe Meikang Information Technology Co., Ltd.” was used to collect medical records. The specific steps were as follows: (1) “pneumonia” was input in “diagnosis”; (2) “2019.02.01–2022.01.31” was input in “time”, combined with the inclusion and exclusion criteria to select the cases that met the requirements for the study.

Statistical procedures were carried out using SPSS version 26.0. Measurement data with a non-normal distribution were expressed as medians and quartiles. The Mann–Whitney *U* test was used to assess the statistical significance of groups. Categorical variables were expressed as frequencies and percentages. Chi-square and continuity correction of Chi-square tests were used to compare groups.

## Results

### General information

A total of 2739 children with CAP were included in our study, with 1507 (55.02%) males and 1232 (44.98%) females for a male to female ratio of 1.2:1. The median age was 3.84 (interquartile range: 1.78–6.98) years (ranging from 29 days to 18 years of age). There were 2364 cases during the pre-COVID-19 period and 375 cases during the post-COVID-19 period. The number of hospitalized children after the pandemic was 84.14% lower. No significant difference in sex distribution was observed (*P* > 0.05). The median age after the outbreak (2.58 years) was 1.5 years younger than the median age before the outbreak (4.08 years), and the difference was statistically significant (*Z* = − 7.885, *P* < 0.001). The proportion of CAP in the infant group increased, and in the school-age group, it decreased significantly (*P* < 0.001). The positivity rate for pathogen detection decreased in spring and summer and increased in autumn and winter during the post-COVID-19 period (*P* < 0.05). A total of 650 patients with chronic diseases were included in this study, including bronchial asthma, bronchiectasis, tracheal malformation, esophageal dilatation, congenital heart diseases, hypertension, epilepsy, nephrotic syndrome, leukemia, hyperthyroidism, allergic purpura, congenital genetic metabolic diseases, immune deficiency and other chronic diseases of various systems. The number of CAP patients with chronic diseases during the post-COVID-19 period was significantly greater (*P* < 0.001) (Table 1).

**Table 1:** The distribution of general data

Groups	Pre-COVID-19 period (*n* = 2364)		Post-COVID-19 period (*n* = 375)	
	Cases of pathogen	Percentage, %	Cases of pathogen	Percentage, %
Sex				
Male	1300	54.99	207	55.20
Female	1064	45.01	168	44.80
Age				
Infant	299^‡^	12.65	89	23.73
Younger age	575	24.32	107	28.53
Pre-school children	670	28.34	111	29.60
School-age children	820^‡^	34.69	68	18.13
Season				
Spring	622^‡^	28.40	31	9.14
Summer	571^‡^	21.07	38	11.21
Autumn	587^*^	26.80	110	32.45
Winter	410^‡^	18.72	160	47.20
Chronic diseases				
Yes	502^‡^	21.24	148	39.47
No	1862	78.76	227	60.53

*COVID-19* coronavirus disease 2019. The Chi-square test was used for the above comparisons; ^*^P < 0.05, ^‡^*P* < 0.001 between the two groups

### Pathogenic distribution

**Table 2:** The distribution of total pathogens

Pathogen species	Cases and percentage, *n* (%)		Chi-square test	
	Pre-COVID-19 period (*n* = 2364)	Post-COVID-19 period (*n* = 375)	*χ* ^2^	*P*
Bacteria	1192 (50.43)	212 (56.53)	4.837	0.028
Gram-positive bacteria	609 (25.76)	117 (31.20)	4.914	0.027
SP	520 (43.62)	79 (37.26)	2.976	0.084
SA	89 (7.47)	37 (17.45)	21.973	< 0.001
Gram-negative bacteria	583 (24.67)	95 (25.33)	0.078	0.779
KP	31 (2.60)	6 (2.83)	0.037	0.848
*E. coli*	29 (2.43)	8 (3.77)	1.216	0.261
HI	468 (39.26)	39 (18.40)	33.965	< 0.001
PA	14 (1.17)	11 (5.19)	14.367	< 0.001
AB	25 (2.10)	13 (6.13)	11.127	0.001
Others	16 (1.34)	19 (8.96)	42.995	< 0.001
Viruses	699 (29.57)	201 (53.60)	84.724	< 0.001
RSV	242 (34.62)	74 (36.82)	0.330	0.566
ADV	154 (22.03)	14 (6.97)	23.340	< 0.001
IV	86 (12.30)	2 (0.10)	22.629	< 0.001
PIV	184 (26.32)	28 (13.93)	13.315	< 0.001
Others	33 (4.72)	83 (41.29)	185.973	< 0.001
MP	1408 (59.56)	88 (23.47)	170.108	< 0.001
CP	13 (0.55)	9 (2.40)	11.679^a^	0.001
Fungi	81 (3.43)	14 (3.73)	0.091	0.763
No pathogen detected	285 (12.06)	70 (18.67)	12.539	< 0.001

*COVID-19* coronavirus disease 2019, *SP Streptococcus pneumoniae*, *SA Staphylococcus aureus*, *KP Klebsiella pneumoniae*, *E. coli Escherichia coli*, *HI Haemophilus influenzae*, *PA Pseudomonas aeruginosa*, *AB Acinetobacter baumannii*, *RSV* respiratory syncytial virus, *ADV* adenovirus, *IV* influenza virus, *PIV* parainfluenza virus, *MP Mycoplasma pneumoniae*, *CP Chlamydia pneumonia*. ^a^The continuous correction Chi-square test

Among the 2739 children with CAP, a total of 2384 pathogenic tests were positive, with 2079 before the pandemic and 305 after the pandemic. The number of CAP cases in children declined substantially post-COVID-19 (Table 2). After the pandemic, the proportion of both bacterial and viral CAP increased, especially viral CAP. Before the onset of COVID-19, the detected pathogenic proportions were as follows: MP (59.56%) > bacteria (50.42%) > viruses (29.57%) > fungi (3.43%). During the post-COVID-19 period, the pathogenic proportions were bacteria (56.53%) > viruses (53.60%) > MP (23.47%) > fungi (3.73%). MP and bacteria were the main pathogens before the onset of the pandemic, and bacteria and viruses were the main pathogens after the onset of the pandemic. The proportions of MP decreased significantly (*P* < 0.001). Gram-positive bacteria were the main bacteria. The top three bacteria before and after the pandemic were *Streptococcus pneumoniae, Haemophilus influenzae* and *Staphylococcus aureus*. In addition, the rates of *Pseudomonas aeruginosa* and *Acinetobacter baumannii* also increased after the outbreak.

There were no significant changes in RSV proportions, and RSV still ranked first. The adenovirus (ADV), influenza virus (IV), and parainfluenza virus (PIV) proportions all decreased after the pre-COVID-19 period, especially IV infection, which nearly became zero. The proportions differed significantly (*P* < 0.001). All patients after the pandemic in this study tested negative for SARS-CoV-2.

### Single pathogen infection and coinfection

The rate of coinfections was higher than that of any single pathogen infection and did not change significantly both before and after pandemic onset. MP was the most common single-detected pathogen in the pre-COVID-19 period, and viruses were the most common in the post-COVID-19 period, followed by MP (Table 3). After the outbreak, the positivity rates of bacteria–MP coinfection decreased. However, the rates of virus–virus and virus–bacteria coinfection increased greatly after the outbreak, and the difference was significant (*P* < 0.001) (Table 4).

**Table 3:** The distribution of one pathogen infection and coinfection

Pathogen species	Cases and percentage, *n* (%)		Chi-square test	
	Pre-COVID-19 period (*n* = 2364)	Post-COVID-19 period (*n* = 375)	*χ*^2^	*P*
Bacteria	178 (7.53)	36 (9.60)	1.926	0.165
Viruses	155 (6.56)	58 (15.47)	35.827	< 0.001
MP	825 (34.90)	50 (13.33)	69.234	< 0.001
CP	2 (0.08)	2 (0.05)	–	0.093
Fungi	6 (0.25)	5 (1.33)	6.924^a^	0.009
Coinfection	913 (38.62)	154 (41.07)	0.814	0.367
Others	285 (12.14)	70 (19.20)	14.163	< 0.001

*COVID-19* coronavirus disease 2019, *MP Mycoplasma pneumoniae*, *CP Chlamydia pneumonia*. ^a^The continuous correction Chi-square test. “–” Fisher’s exact test

**Table 4:** The distribution of coinfection

Groups	Cases and percentage, *n* (%)		Chi-square test	
	Pre-COVID-19 period (*n* = 913)	Post-COVID-19 period (*n* = 154)	*χ* ^2^	*P*
Virus + virus	18 (1.97)	14 (9.09)	20.577^a^	< 0.001
Bacteria + virus	184 (20.15)	71 (46.10)	48.792	< 0.001
Bacteria + bacteria	86 (9.42)	22 (14.29)	3.430	0.064
Virus + MP	147 (16.10)	18 (11.69)	1.963	0.161
Bacteria + MP	307 (33.63)	11 (7.14)	44.174	< 0.001
Bacteria + virus + MP	91 (9.97)	7 (4.55)	4.644	0.031
Others	80 (8.76)	11 (7.14)	0.443	0.506

*COVID-19* coronavirus disease 2019, *MP* *Mycoplasma pneumonia*. ^a^The continuous correction Chi-square test

## Discussion

The number of children hospitalized with CAP during the post-COVID-19 period was 84.14% lower than the number hospitalized during the pre-COVID-19 period, especially among school-age children. We inferred that the decline after the outbreak was attributed to the strict prevention and control measures implemented in Beijing, such as hand hygiene practices, wearing surgical masks when in crowded places, maintaining a social distance of more than one meter, measuring body temperature and discouraging students with respiratory symptoms from going to school. All of the above factors protected children from infection, especially school-age children.

The reason for the decline in CAP in children in the spring and summer after the pandemic is that the pandemic situation was severe at that time. Beijing launched a high-level emergency response and implemented various measures, such as encouraging home isolation, measuring body temperature, checking access cards, scanning health codes and trip codes, and so on to reduce personal activities and the spread of the disease.

In addition, our study showed that the age of children suffering from CAP decreased by 1.5 years after the onset of the pandemic. We believe that this was because the proportion of CAP in school-age children decreased significantly, but the proportion in infant and younger children did not. For the same reason, the proportion of CAP patients without chronic disease decreased significantly, but the proportion of CAP patients with chronic disease did not. Infants, younger children and children with chronic diseases had hypoimmunity. Despite the above protective measures, these children were still more susceptible to infection than school-age children and children without chronic diseases. Another reason for the higher proportion of children with chronic diseases was that they tended to be sicker after infection, and parents were more motivated to seek medical attention.

In our study, a pathogen was detected in 2079 of the 2364 children (87.94%) during the pre-COVID-19 period and in 305 of the 375 children (81.33%) during the post-COVID-19 period, which differed from some other studies [5]. This difference may be attributed to the year of analysis and to differences in the populations and countries studied.

In our study the positivity rate of MP decreased from 59.56% to 23.47%; furthermore, the positivity rates of ADV, IV, and PIV all decreased during the post-COVID-19 period, especially influenza virus pneumonia, which was nearly zero. Many reports before the outbreak showed that MP was the most common pathogen of CAP in children. Atkinson et al. [6] showed that 40% of pediatric CAP in children greater than five years of age was caused by MP. Research shows that MP bacteria cause an epidemic every 3–5 years and may persist for 1–2 years [7–9]. The reduction in MPP may be affected by the epidemic year to some extent, but we still believe that it was mainly due to the mitigation measures that rates of MPP were so drastically reduced. In addition, IV is a common pathogen of CAP in children in the winter season, and studies have shown that IV also easily causes outbreaks on school campuses and in kindergartens [10, 11]. The mitigation measures were beneficial to restrain the spread of MP and IV in the first place. RSV was still the most common pathogen for virus infection, which was consistent with most reports in China and abroad [12–14].

The experiment also showed that Gram-positive bacteria were the main strain detected before and after pandemic onset, and the top three bacteria were *Streptococcus pneumoniae, Haemophilus influenzae* and *Staphylococcus aureus*, which was in accordance with the results of Nathan et al. [15] and Mathew [16] but was inconsistent with the results of Ji et al. [17] and Liu et al. [18], who reported that Gram-negative bacteria were the most common agents. *Klebsiella pneumoniae* was the most common bacteria. The reasons may be related to the region, time and climate. The increased proportions of *P. aeruginosa-* and *A. baumannii-*associated CAP after the outbreak may be related to the increased proportion of CAP patients with chronic disease.

This study showed that the positivity rate of coinfections was higher than that of any single pathogen before and after the onset of the pandemic, which was much higher than the 29% reported in the study by Juvén et al. [19]. The reason may be that different countries, sample sizes, times and other factors may have an impact on the distribution of pathogens. Coinfection is more likely to induce serious inflammation and clinical diseases [20, 21]; so coinfection should be considered when the condition of a patient is serious.

A limitation of this study is that it included only a single pediatric center. We look forward to more relevant reports to provide a basis for developing countries to formulate pediatric CAP prevention and control measures.

In conclusion, there was a significant decrease in the number of children hospitalized with CAP after the pandemic, especially among school-age children, and the pathogenic proportions of CAP with MP and IV were significantly decreased. We inferred that CAP was effectively prevented in school-age children because of the strong mitigation measures. This may help health managers, medical workers and educators develop policies and measures to prevent CAP in the future.

## Data Availability

The datasets generated during and analysed during the current study are available from the corresponding author on reasonable request.
